# Paediatric lung injury due to accidental ingestion of meperfluthrin: a case report

**DOI:** 10.1186/s12887-022-03117-4

**Published:** 2022-01-17

**Authors:** Zhongqiang Li, Xuejun Wu, Gaomei Lv, Zhijuan Ren, Huimin Yang, Leilei Xu, Qingli Guan, Xiuqi Meng

**Affiliations:** Department of Pediatrics, Linyi People’s Hospital, Linyi, 276000 Shandong Province China

**Keywords:** Meperfluthrin, Inflammatory reaction, Lung injury, Pyrethroid pesticide, Poisoning

## Abstract

**Background:**

It is common for children to accidentally ingest chemical drugs with different degrees of toxicity. Meperfluthrin is a highly effective and easy-to-use pyrethroid pesticide with low toxicity. It is widely used in electric mosquito coils. This type of electric mosquito coil is used in daily life, which increases the chance of exposure among children and, consequently, may lead to accidental ingestion. There are only few reports of meperfluthrin poisoning causing lung injury in children. We report a rare clinical case of lung injury wherein a child ingested meperfluthrin orally.

**Case presentation:**

We report the case of a 1-year-old boy who accidentally swallowed an electric mosquito coil containing meperfluthrin and developed cough and fever. The patient’s parents observed him swallowing the electric mosquito coil (Qiangshou®). Although he was stopped, the child had already swallowed approximately 10 ml of the liquid. According to the instructions, it contained 9 mg/ml of meperfluthrin, thus, it was assumed that he ingested meperfluthrin at a dose of approximately 90 mg. Computed tomography (CT) of his lungs showed uneven brightness in both lungs with multiple spots, scaly shadows, and mesh. Density of the shadows indicated lung parenchymal and interstitial lung disease. Lung tidal function tests indicated obstructive ventilation dysfunction. After evaluation and treatment, his cough drastically reduced, his fever disappeared, and his lung CT findings showed improvement. Therefore, accidental ingestion of meperfluthrin led to acute lung injury in a paediatric patient. Because of prompt treatment, his lung lesions recovered well.

**Conclusions:**

Meperfluthrin causes airway mucosal damage and hypersensitivity. Lung CT and lung tidal function measurements can be used to monitor changes in the condition. Presently, there is a lack of specific detoxification drugs for meperfluthrin poisoning. Thus, the focus of treatment is to protect the airway mucosa and reduce inflammatory reactions.

## Background

Poisoning caused by pyrethroid pesticides affects the nervous system, characterised by symptoms such as headache, dizziness, coma, convulsions, and blurred vision. The manifestations of respiratory system damage include cough, chest tightness, difficulty in breathing, and sore throat. Other symptoms include oral ulcers, gastroesophageal ulcers, erosive gastritis, nausea, vomiting, dysphagia, abdominal pain, arrhythmia, skin burning, itching, rash, and other injuries [1]. Meperfluthrin is a highly effective and easy-to-use pyrethroid pesticide with low toxicity. It is widely used in electric mosquito coils. This type of electric mosquito coil is used in daily life that increases the chance of exposure among children and, consequently, may lead to accidental ingestion. Thus far, there are only few reports of meperfluthrin poisoning causing lung injury in children. Here, we report a rare clinical case of lung injury in a child caused by the ingestion of meperfluthrin.

## Case presentation

**Fig. 1:**
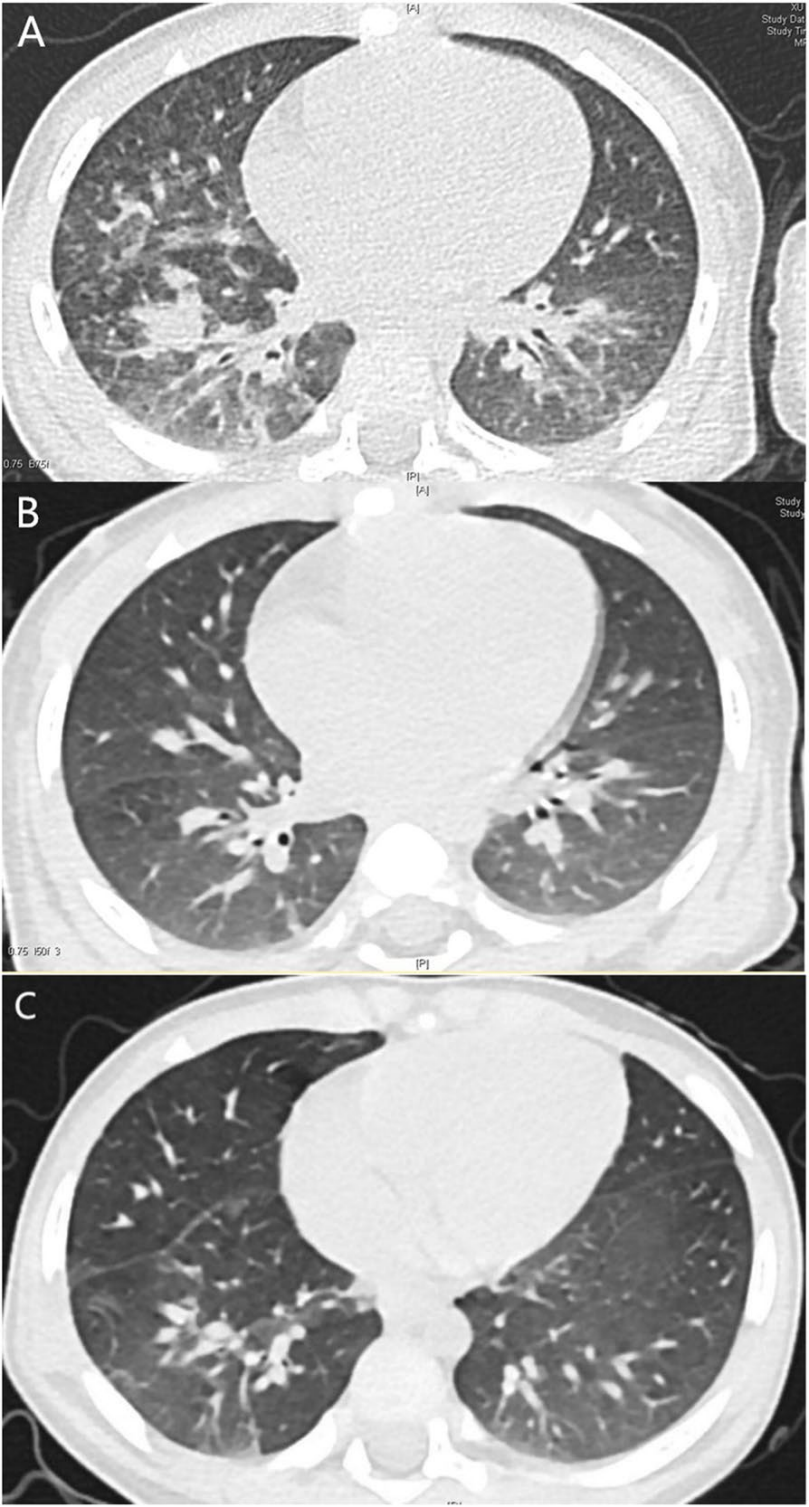
**A** Chest computed tomography (CT) scan. Image showing ground-glass shadows, diffuse grid-like shadows, multiple patchy shadows, thickened lung septum, and uneven brightness enhancement in both lungs on day 4. **B** Chest computed tomography (CT) scan. Image showing uneven brightness of the lungs along with multiple patchy shadows, blurred borders on day 10. **C** Chest computed tomography (CT) scan. Image showing reduced lung patchy shadows after treatment on day 50

A 1-year-old boy accidentally swallowed meperfluthrin. He had no respiratory disease symptoms for the first 3 days. On the fourth day, he developed cough, at a frequency of 4–5 times a day, accompanied by sputum, and no wheezing. No dyspnoea or skin cyanosis was observed. Then the patient is hospitalized for treatment. On physical examination, bilateral pupil diameter was 3 mm and reactive to light. Thickened breathing and sputum sounds could be heard on lung auscultation. Other clinical parameters included respiratory rate of 28 breaths per minute and heart rate of 132 beats per minute; blood pressure was 79/46 mmHg. Initial arterial blood gas analysis indicated pH 7.418, pO2 93.5 mmHg, pCO2 34.7 mmHg, HCO3 22 mmol/L, base excess − 1.5 mmol/L, plasma lactate 1.6 mmol/L. Serum electrolytes, lactate dehydrogenase, creatine phosphokinase, alanine aminotransferase, aspartate aminotransferase, and creatinine level were normal. Routine blood tests were performed, with a normal C-reactive protein (CRP) level; and electroencephalogram showed no abnormality; left ventricular ejection fraction is 66%. Computed tomography (CT) of the lungs showed ground glass shadows, diffuse grid-like shadows, multiple patch-like shadows, thickened pulmonary septa, and uneven brightness in both lungs (Fig. 1A). Under quiet sleep conditions, lung moisture function was measured using the German Jaeger Pulmonary Function Instrument. The results showed that the tidal volume was 9.2 mL/kg, the respiration rate was 25.7 breaths per min, the inhalation time was 0.88 s, the exhalation time was 1.46 s, and the inhalation-to-exhalation ratio was 0.6. The peak time ratio was 12.2%, and the peak volume ratio was 17.7%. Therefore, moderate-to-severe obstructive ventilatory dysfunction was considered. Lung CT findings indicated pulmonary inflammatory exudation. Lung moisture function test indicated small airway obstruction.

The patient was recommended to perform inhalation of budesonide suspension to reduce small airway inflammatory exudation and atomized inhalation of terbutaline to expand small airway treatment after hospitalization. Budesonide suspension 1 mg and terbutaline nebulized liquid 2.5 mg each time, twice daily. Fever occurred on day 6. Body temperature fluctuated between 37 °C and 40 °C. Pupil size was normal, blood pressure was stable, breathing and pulse were stable, laboratory test blood routine and CRP were normal. There was cough with sputum, and therefore, lung inflammation was considered. On day 10, cough, fever, and lung phlegm disappeared, and lung CT showed improved lesions and shadow area absorption (Fig. 1B). The child was discharged from the hospital, and home observations were continued.

On day 50, the child had no cough or fever, and the lung CT condition had improved (Fig. 1C). In lung moisture function measurements, the tidal volume was 9.9 mL/kg, respiratory rate was 27.9 breaths per min, inhalation time was 0.79 s, exhalation time was 1.36 s, and the inhalation-to-exhalation ratio was 0.58. The peak time ratio was 21%, and the peak volume ratio was 24.4%, suggesting moderate-to-severe obstructive ventilatory dysfunction. No symptoms had occurred after 12 months of follow up. The lung function had returned to a normal range for the patient’s age group.

## Discussion and conclusions

Accidental drug poisoning in children is a global public health problem worldwide. Poisoning not only seriously affects children’s life, health, growth, and development but also imposes a heavy burden on their families and social medical care [2]. Reports have shown that the incidence of poisoning among children under the age of 18 in the emergency room is 0.47%, and the highest rate of drug poisoning is 42.7% [3]. Pyrethroid pesticides are a class of synthetic biomimetic pesticides with high insecticidal activity. These pesticides have lower toxicity than organophosphorus pesticides. Most pyrethroid pesticides have low to medium toxicity. Dietary, respiratory, and cutaneous contact is the most common modes of human exposure to pyrethroid pesticides [4]. Meperfluthrin is a highly effective fluorine-containing insecticide with low-toxicity. It is used in the manufacture of electric mosquito coils. Here, we have described a case of a child who accidentally ingested an electric mosquito coil containing meperfluthrin. Unfortunately, no blood or urine was sent for analysis of metabolites of meperfluthrin, due to the lack of a lab for toxins identification. Because of cost reasons, the parents refused to send specimens for identification of serum toxicant concentration.

Fluorinated insecticides are highly fat-soluble and hydrophobic which improve their absorption and transport rates within organisms. Studies have shown that a variety of pathogenic mechanisms, such as tissue cell hypoxia, cytotoxicity, and damage to alveolar surfactants, may contribute to the pathophysiology of pyrethroids. Pyrethroids target the nervous system and change the sensitivity of sodium ion channels in the nerve membrane. Therefore, the channels remain open for a prolonged duration, and sodium ions flow continuously. Consequently, action potential depolarization is prolonged, and nerve cells are abnormally excited. The concentration of the drug determines the proportion of sodium channels opened and the duration for which they remain open [1]. Moreover, pyrethroids target the endothelium of alveolar capillaries, causing extensive cell damage, increasing the permeability of the capillary wall, and leading to the accumulation of a large amount of fluid in the lung interstitium and alveoli, ultimately resulting in pulmonary oedema [5]. The toxin combines with cytochrome oxidase to inhibit the electron transfer of the cell’s respiratory chain. Therefore, the cell loses the ability to use oxygen for oxidative phosphorylation and energy generation, resulting in hypoxia of tissue cells, increased capillary wall permeability, and pulmonary oedema [6]. In addition, alveolar surfactant damage is one of the causes of pulmonary oedema caused by pyrethroids. These drugs can damage the sub-microscopic structure of lung cells and alveolar capillaries, which leads to increased pulmonary capillary permeability. Both type I and type II alveolar epithelia have inward-transport sodium channels. Because pyrethroids can affect sodium channels and destroy osmotic gradients, they can cause airway and bronchial mucosal oedema [7]. Furthermore, pyrethroids can cause hypersensitivity leading to inflammation [1]. In addition, the inhibitory effect of toxins on intracellular respiration can cause extensive hypoxic damage to the myocardium, affecting cardiac function [8],cardiac hypotrophy contributing to the formation of pulmonary oedema. Brain dysfunction caused by cerebral hypoxia, cerebral oedema, and increased intracranial pressure can cause neurogenic pulmonary oedema [9, 10].

Pulmonary oedema caused by toxins appears as ground glass opacity or consolidation on CT scan, which may be diffuse or focal. In this case, lung CT findings showed inflammatory exudation of the lung, pathological changes in the lung parenchyma and interstitium, and fever during the course of the disease. Moreover, inflammatory reactions were considered. In this case, the child mistakenly swallowed meperfluthrin. On day 4, he developed cough. On day 6, he developed fever. Lung imaging showed double lung exudate. Blood test results and CRP levels were normal. These observations suggested lung injury due to the effect of the drug on the sodium channel. However, there was insufficient evidence to suggest secondary lung infection.

Meperfluthrin causes airway mucosal damage and hypersensitivity. Lung CT and lung tidal function measurements can be used to monitor changes in the condition. Presently, there is a lack of specific detoxification drugs for meperfluthrin poisoning. Thus, the focus of treatment is to protect the airway mucosa and reduce inflammatory reactions. It is necessary to strengthen the supervision of children and improve safety awareness. Additionally, electric mosquito coil liquids should be stored properly to avoid contact. This is key to effectively reducing lung injury caused by poisoning in children.

### Availability of data and materials

The data that support the findings of this case report are available from the corresponding author upon reasonable request.

## Abbreviations

CT: Computed tomography
CRP: C-reactive protein
